# CoDiab-VD: protocol of a prospective population-based cohort study on diabetes care in Switzerland

**DOI:** 10.1186/s12913-015-0991-0

**Published:** 2015-08-14

**Authors:** Emilie Zuercher, Julie Bordet, Bernard Burnand, Isabelle Peytremann-Bridevaux

**Affiliations:** Institute of social and preventive medicine (IUMSP), Lausanne University Hospital, Lausanne, Switzerland

## Abstract

**Background:**

Diabetes represents an increasing health burden worldwide. In 2010, the Public Health Department of the canton of Vaud (Switzerland) launched a regional diabetes programme entitled “Programme cantonal Diabète” (PcD), with the objectives to both decrease the incidence of diabetes and improve care for patients with diabetes. The cohort entitled CoDiab-VD emerged from that programme. It specifically aimed at following quality of diabetes care over time, at evaluating the coverage of the PcD within this canton and at assessing the impact of the PcD on care of patients with diabetes.

**Methods/Design:**

The cohort CoDiab-VD is a prospective population-based cohort study. Patients with diabetes were recruited in two waves (autumn 2011 - summer 2012) through community pharmacies. Eligible participants were non-institutionalised adult patients (≥18 years) with diabetes diagnosed for at least one year, residing in the canton of Vaud and coming to a participating pharmacy with a diabetes-related prescription. Women with gestational diabetes, people with obvious cognitive impairment or insufficient command of French were not eligible. Self-reported data collected, included the following primary outcomes: processes-of-care indicators (annual checks) and outcomes of care such as HbA1C, (health-related) quality of life measures (Short Form-12 Health Survey – SF-12, Audit of Diabetes-Dependent Quality of Life 19 – ADDQoL) and Patient Assessment of Chronic Illness Care (PACIC). Data on diabetes, health status, healthcare utilisation, health behaviour, self-management activities and support, knowledge of, or participation to, campaigns/activities proposed by the PcD, and socio-demographics were also obtained. For consenting participants, physicians provided few additional pieces of information about processes and laboratory results.

Participants will be followed once a year, via a mailed self-report questionnaire. The core of the follow-up questionnaires will be similar to the baseline one, with the addition of thematic modules adapting to the development of the PcD. Physicians will be contacted every 2 years.

**Discussion:**

CoDiab-VD will allow obtaining a broad picture of the care of patients with diabetes, as well as their needs regarding their chronic condition. The data will be used to evaluate the PcD and help prioritise targeted actions.

**Trial registration:**

This study is registered with ClinicalTrials.gov, identifier NCT01902043, July 9, 2013.

**Electronic supplementary material:**

The online version of this article (doi:10.1186/s12913-015-0991-0) contains supplementary material, which is available to authorized users.

## Background

Worldwide, chronic diseases constitute a major burden for communities in terms of morbidity, disability and mortality. Their care requires collaboration between healthcare providers, teamwork, training in self-management and the use of evidence based-medicine. However, such care processes are complex and often suboptimal. Thus, chronic disease management (CDM) strategies have been developed as a means of reorganizing healthcare systems and medical treatment for chronic diseases [1–4].

Diabetes is one of the most common chronic diseases, with an estimated number of affected persons of more than 550 million for 2030 [5]. In the canton of Vaud, a Swiss state of ~720.000 residents (approximately 10 % of the Swiss population), a recent population-based study has shown a prevalence of diabetes of about 7 % [6]. In 2010, the Public Health Department of the canton of Vaud initiated the development of a regional programme entitled “Programme cantonal Diabète” (PcD), with both the aim to decrease the prevalence of diabetes in that canton and improve care for patients with diabetes [7]. The political will and support for this innovative programme, at the level of a whole canton, is unique in Switzerland.

Evaluation of CDM programmes is essential to assess their implementation and determine the benefits and effectiveness of their development [8, 9]. Randomized controlled trials (RCT) may not be the most suitable design for evaluating complex interventions such as the PcD, since such interventions are very much context-dependent [10]. In addition, there might be practical difficulties to include control sites, and the resources and costs incurred may be substantial. Moreover, when implemented at a macro level, with no direct inclusion of patients, RCT cannot be considered as an evaluation alternative. Scientifically sound evaluation methods that remain also practical in routine settings are needed; cohort studies may represent such an opportunity.

To assess the impact of the implementation of the PcD on health and care outcomes as well as to obtain a comprehensive picture of patients with diabetes residing in the canton of Vaud, and their care, we opted for a prospective cohort study design. After the baseline data collection, participants will be followed-up annually to assess the evolution of the quality of care over time, the coverage of the PcD within that canton and the impact of the PcD on patients’ care.

## Methods/Design

### Design

**Fig. 1:**
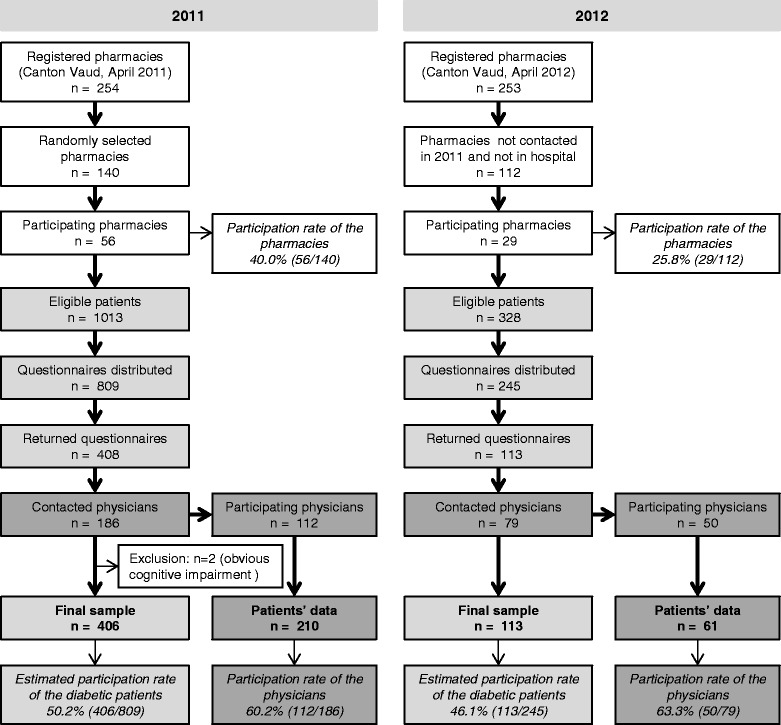
Recruitment flowchart ot the CoDiab-VD cohort

**Fig. 2:**
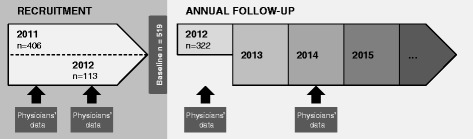
Study design of the CoDiab-VD cohort

The CoDiab-VD is a prospective population-based cohort study launched in 2011 in the canton of Vaud/Switzerland, conducted by the Institute of Social and Preventive Medicine (IUMSP) of the Lausanne University Hospital. Baseline data were collected during a two-wave recruitment period in the autumn of 2011 and in the summer of 2012 (Fig. 1). Patients’ follow-ups will be set up yearly (Fig. 2).

### Study population

#### Sampling frame

Randomly selected community-based pharmacies registered in the canton of Vaud were contacted and asked to participate in the recruitment of patients with diabetes [11]. During a 6 week period, participating pharmacies had to propose the survey to 40 consecutive persons visiting the pharmacy with a prescription for diabetes-related treatment and/or equipment (oral anti-diabetic medications, insulin, glycemic strips or glucose meter). Pharmacists checked patients’ eligibility and briefly described the context and aims of the study. The baseline questionnaire package (information letter, questionnaire, prepaid reply envelope) was given to eligible patients accepting to take it, to complete it at home, and to return it to the investigators by regular mail. Pharmacists did not record contact details of eligible participants because of time and organisation constraints; therefore potentially participating patients with diabetes could not be reminded to send back the questionnaire. For the same reason, the characteristics of non-participants could not be recorded.

### Inclusion criteria

Patients were eligible if they came to a participating pharmacy with a diabetes-related prescription, and were non-institutionalised adults (≥18 years) reporting a diagnosis of diabetes for at least 1 year, residing in the canton of Vaud and able to provide written informed consent.

### Exclusion criteria

Exclusion criteria at baseline were gestational diabetes, obvious cognitive impairment, or insufficient level of French to understand and complete the self-report questionnaire.

### Sample size

The number of patients with diabetes to include in the study was estimated to obtain good precision (i.e. confidence interval width) around the following primary outcomes at baseline: mean HbA1C value, mean Physical and Mental component scores (PCS and MCS) of the Short Form-12 Health Survey (SF-12), mean Patient Assessment of Chronic Illness Care (PACIC) global score, and percentage of patients receiving recommended annual processes-of-care. Considering the clustering of data by pharmacies (40 pharmacies, each recruiting 15 patients, intra-class correlation 0.05, alpha 0.05, beta 0.2), the recruitment of 600 participants was deemed appropriate. This sample size was also large enough to detect a 0.5 % point decrease in HbA1C between two time points. In addition, it was above the sample size required for such a change considering repeated measures within a same individual.

### Baseline measures

#### Patients’ questionnaire

The baseline questionnaire was designed to encompass several aspects of disease, care and life of people living with diabetes. It targeted the following areas: diabetes status, diabetes management and quality of care, health-related quality of life (HRQoL) and quality of life (QoL), health services utilisation, health status and health habits, self-management activities and support, socio-demographics. An overview of all variables collected is shown in Table 1, and a comprehensive description of corresponding questionnaires and answer categories is provided in the Additional file 1.

**Table 1:** Overview of the variables, measures and instruments used in the patient self-completed questionnaire

Section	Themes	Instruments, details
Primary outcomes: processes and outcomes quality of care indicators		
Diabetes management	Receipt of recommended processes-of-care	1) Past 12 months: HbA1C check, blood pressure measurement, weight measurement, lipid profile, diabetic foot examination, urine test for microalbuminuria, eye examination by ophtalmologist, influenza vaccination
		2) Anytime: physical activity recommendations, diet recommendations
	HbA1C	Last HbA1C value
Health-related quality of life (HRQoL) and Quality of life (QoL)	Generic HRQoL	SF-12 [15]
	Diabetes-specific QoL	ADDQoL [16]
Patient assessment of chronic care	Congruency of care with the Chronic Care Model (CCM)	PACIC [17, 18]
Exposure variables		
Programme cantonal Diabète^a^	Knowledge of/participation in PcD activities/projects	
Other variables of interest		
Diabetes	Characteristics of the disease	Type of diabetes, disease duration, treatment (drugs)
	Diabetes-related complications	List of following complications: ischemic heart diseases, stroke, retinopathy, chronic kidney disease (CKD) without dialysis, CKD with dialysis or kidney transplant, neuropathy, foot ulcer, lower limb amputation, severe hypo- or hyperglycemia
Diabetes management	Medication adherence	Morisky Medication Adherence Questionnaire [19]
Health services utilisation	Ambulatory care visits, emergency visits, hospitalisation	Utilisation during past 12 months
	Home care services, domestic home support	Received help during past 12 months
	Foregoing care because of costs	Foregoing care during past 12 months
Health status and health habits	Anthropometric values	Weight, height
	Smoking	Smoking status, duration of smoking, smoking products, average number of cigarettes smoked per day, medical advice on smoking cessation
	Alcohol consumption	AUDIT-C questionnaire [20]
	Physical activity levels	Questions from the Swiss Health Survey [21]
	Depression screening	Two validated questions for the screening of depression [22]
	Comorbidities	List of following chronic diseases: heart disease (heart failure, valve disease, heart muscle disease), chronic lung disease (asthma, chronic bronchitis, emphysema), osteoporosis, osteoarthritis or arthritis; cancer or malignancy or lymphoma (with the exception of skin cancer), gastric or duodenal ulcer, depression, Parkinson disease, hypertension, hyperlipidemia, other chronic condition
Self-management activities and support	Home glucose self-monitoring	
	HbA1C knowledge	
	Participation in diabetes education courses	
	Membership in the local diabetes association	
	Knowledge and use of the “Diabetes Passport”^b^	
	“Self-efficacy”	Level of easiness/difficulty to manage diabetes generally, and regarding physical activity, diet, and medication
	Level and source of information about diabetes	
	Support and satisfaction from healthcare team and social network	
	Diabetes care satisfaction and recommandation of their care to others	
Socio-demographics	Characteristics of the participants	Age, gender
	Socio-economic status	Marital status, family size, household income, education, employment, insurance status, place of residence, nationality

*HRQoL* health-related quality of life, *SF-12* short form-12 Health Survey, *ADDQoL* audit of diabetes-dependent quality of life 19, *CCM* chronic care model, *PACIC* patient assessment of chronic illness care

^a^Since the 2012 recruitment

^b^A small booklet with data, information and reminders

#### Selection of indicators

The selection of quality indicators followed a three-step procedure. First, we searched the published and grey literature for frequently used diabetes quality of care indicators and population-based surveys exploring the quality of care of patients with diabetes. Then, we identified published clinical practice guidelines on diabetes care and considered those most recently updated and used by a group of regional partners adapting diabetes guidelines for Switzerland. The guidelines developed by the following agencies were considered: National Institute for Health and Clinical Excellence (NICE), Scottish Intercollegiate Guidelines Network (SIGN), International Diabetes Foundation (IDF), Canadian Diabetes Association (CDA), American Diabetes Association (ADA), Haute Autorité de Surveillance (HAS). Finally, we took into account three criteria: clinical significance, practical relevance (feasibility), and reliability/validity of the measurement. When several instruments existed, we considered their validity, their length, and the existence of a French version. We favoured instruments which presented the best balance of those latter criteria.

#### Primary outcomes: processes and outcomes quality of care indicators

We considered as primary outcomes all usually recommended diabetes processes-of-care indicators, as well as the following outcomes of care indicators: HbA1C levels, as an intermediary outcome associated with future development of diabetes complications [12–14], HRQoL and QoL as measured using a generic tool (SF-12) [15] and a diabetes-specific quality of life tool (Audit of Diabetes-Dependent Quality of Life 19 – ADDQoL) [16], respectively, and patient assessment of chronic care, i.e. how care is congruent with the Chronic Care Model (PACIC) [17, 18].

#### Exposure variables

In the 2012 baseline questionnaire, we also added a small number of questions regarding knowledge and participation in the few activities proposed that year by the PcD. These questions were only asked to patients recruited in 2012 in order to describe possible differences in terms of exposition to, and awareness of, the PcD, because patients were recruited over two different time periods.

#### Other variables of interest

The other variables of interest considered in the baseline questionnaire are briefly described thereafter; more details are provided in Table 1 and Additional file 1.

- Diabetes characteristics and related complications;
- Medication adherence using the Morisky medication adherence questionnaire [19];
- Healthcare utilisation within the past 12 months: ambulatory care visits, emergency visits, hospitalisation, home care services, domestic home support;
- Care foregone because of costs;
- Health status and health habits: anthropometric measures (weight and height allowing the calculation of the body mass index (BMI)), smoking status, alcohol consumption using the AUDIT-C questionnaire [20], levels of physical activity using questions from the Swiss Health Survey [21], depression screening using two validated questions [22] and comorbidities;
- Self-management activities and support measures: home glucose self-monitoring, HbA1C knowledge, participation in diabetes education classes, membership of the local diabetes association (Association Vaudoise du Diabète – AVD), knowledge of the “Diabetes Passport” (a small booklet with data, information and reminders) and if it was known, whether it was used;
- “Self-efficacy” measure, which was developed de novo because available instruments were either not appropriate from our point of view, or too long or did not have a French version. We were interested in exploring how easy/difficult it was for patients to manage their diabetes, overall, and also specifically, regarding the daily management of physical activity, medication and diet;
- Level and source of information about diabetes;
- Support and satisfaction from the healthcare team or from the members of the social network, if any;
- Overall satisfaction with current care and care recommendation to others;
- Patients’ socio-demographic characteristics: age, gender, nationality, place of residence, marital status, family size, education, employment, household income and insurance status.

#### Pretest of the questionnaire

Before the recruitment, we pretested the questionnaire among 12 patients with diabetes to ensure the understanding and acceptability of the instructions and of the questions, as well as to measure the completion time of the questionnaire as a whole.

### Treating physicians’ questionnaire

If participants agreed to share their physicians’ contact details, the physicians were contacted and asked to complete a questionnaire on their patients’ clinical, laboratory and processes-of-care values, as well as a personal questionnaire including some of their characteristics and characteristics of their practice (Table 2).

**Table 2:** Overview of the variables, measures and instruments used in the Physician-completed questionnaires

Section	Variables	Details/Answers
*Patients’ information*		
Diabetes	Diabetes type	
Laboratory results	HbA1C, lipid profile, serum creatinine^a^, urine microalbuminuria, blood pressure, weight, height	Last value and date
Processes-of-care	Diabetic foot examination, eye examination by ophtalmologist, influenza vaccination	Done/not done and date
Global satisfaction with patient managment	Satisfaction with patients’ care and management, barriers to better care management	
*Physicians’ characteristics*		
Personal information	Age, gender, year of diploma, year of start of private practice, board certification type	
Practice information	Practice location, practice type, activity rate, participation in quality programmes/circles	

^a^Since the 2012 follow-up

#### Patients’ information

Physicians were asked to report the type of diabetes (type 1, type 2, other:specify), the last value and date of the following measures: HbA1C, lipid profile, serum creatinine (since the 2012 follow-up), urine microalbuminurie, blood pressure, weight and height. They were also asked to report whether the following processes-of-care were done or not as well as the date of the examination: foot examination, eye examination by ophthalmologist, influenza vaccination. Finally, they had to assess satisfaction with their patients’ care and management (5-point scale: entirely satisfied to not at all satisfied), as well as describe the barriers they were facing which prevented them to better take care of their patients (no barrier, insufficient time during consultation, other health problems of the patient, lack of motivation of the patient, health insurance reimbursement or insurance problem, language or cultural barrier, other).

#### Physicians’ characteristics

Physicians’ characteristics encompassed age, gender of the physician, year of medical diploma, year of start in private practice, and board certification type. We also asked a few questions allowing the characterization of their practice: location and type, activity rate, participation in quality programmes/circles.

### Follow-up

#### Follow-up process

Participants will be followed annually by mail questionnaires (information letter, paper questionnaire and prepaid reply envelope). To maximise retention of participants in the cohort, we will send a postcard reminder 2 weeks after the first mailing to non-respondents and a complete reminder package again 2 weeks later (questionnaire, information letter and prepaid reply envelope). Persistent non-respondents will be contacted by telephone; a minimum of three contact attempts over different days and times is planned.

Because of time constraints and overall limited participation to research projects, treating physicians will be contacted every 2 years only.

### Follow-up questionnaires

The core of the follow-up patients’ questionnaires will be similar to the baseline questionnaire, particularly with regard to the questions targeting primary outcomes, healthcare utilisation, health status and health habits as well as to the questions assessing knowledge and participation to PcD projects. According to the needs and specific interests of the PcD, new questions or thematic modules will be added to the follow-up questionnaires. In 2013 for example, a module on diabetic foot, as well as the Stanford self efficacy questionnaire and a health literacy question were added. In order to keep a questionnaire of a reasonable length, questions representing variables other than primary outcomes may be removed momentarily from the follow-up questionnaire.

The physicians’ questionnaire will be similar to the baseline one.

### Data entry and analysis

Upon receipt, questionnaires are first checked for obvious mistakes, and then scanned with an automated forms processing system, TeleForm™. Exported data are then systematically verified to detect errors. Patients’, physicians’ and pharmacies’ administrative data are kept in separate tables. A unique ID allows to link clinical and administrative information.

Descriptive analyses (univariate and bivariate) will be performed first. Then, depending on the research questions, appropriate statistical analyses will be conducted. Analyses, when appropriate, will take into account the hierarchical structure of the data (clustering by pharmacy and longitudinal design).

### Ethical considerations

The study protocol was approved by the Cantonal Ethics Committee of Research on Human Beings of the Canton of Vaud (Protocol N° 151/11). This study is registered with ClinicalTrials.gov, identifier NCT01902043. Informed consent was obtained from all participants, and data will be kept anonymous.

## Discussion

The implementation of CoDiab-VD followed a qualitative study on the evaluation of patients’ and professionals’ needs regarding the management of diabetes in this canton [23]. Both these first qualitative results and CoDiab-VD baseline quantitative results were, and will be useful for the adaptation of the PcD to the needs of healthcare professionals caring for patients with diabetes and other stakeholders. In addition, CoDiab-VD will be a helpful tool i) for the pragmatic evaluation of the PcD, and ii) for the future development and implementation of PcD projects.

One of our study design’s strength is the recruitment through community pharmacies [11], which allowed us to obtain a sample probably more representative of the population of patients with diabetes than a recruitment through medical practices or hospitals. In fact, we hypothesized that this method would limit the selection of patients on the basis of the level of care received, this information not being available to pharmacists. However, this hypothesis could not be confirmed by a comparison of participants’ and non-participants’ characteristics because data on non-participants could not be collected. Elements in favour of an acceptable representativeness are the fact that our study participants did not differ significantly from participants with diabetes of the CoLaus study [6], another population-based study conducted in the same region, regarding a few common characteristics (age, gender, education, smoking status, BMI) (P. Marques-Vidal, personal communication). The minor differences probably stemmed from the fact that the population of the latter cohort were limited to a narrower age range (35–75). Another positive aspect of the cohort CoDiab-VD is the fact that we collected at baseline, and plan to collect in the future, a broad range of quality indicators that include not only commonly considered processes-of-care but also a variety of patient-reported outcomes. In fact, the latter encompass different aspects of diabetes and diabetes care that are important to patients but nevertheless often neglected despite the fact that they represent important measures to take into account when wishing to capture the complexity of the quality of diabetes care [24–26].

The cohort CoDiab-VD is, however, subject to several limitations. First, the number of patients recruited was below the sample size calculated (inclusion of 519 participants instead of the planed 600). However, because of a greater number of clusters (pharmacies) than expected and a conservative sample size calculation, the precision around point estimates was nevertheless acceptable. This sample size is also large enough (power > 90 %) to detect a 0.5 % point decrease in HbA1C, a PCS or MCS change of 5 points, a PACIC change of 0.3, or an absolute change of process of care of 10 %. Second, one could criticize the choice of self-reported data for the majority of the outcomes, since they may be prone to recall bias – an inherent limitation of such data collection, or be over- or underestimated. Specific analyses, on a fraction of the cohort data, demonstrated a good agreement between patient- and physician-reported outcomes for simple processes-of-care, such as measurement of blood pressure, HbA1C, weight and lipid profile [27]. Physician-reported outcomes were asked for in a second phase. Since their collection was dependent on the physicians’ willingness to participate, these variables were only available for a fraction of the sample, about 50 % at both recruitments and first follow-up. We will nevertheless favour the collection of some physician-reported measures (i.e. blood pressure, HbA1C value) in the future because they are more accurate. Third, between the 2011 baseline recruitment and their first follow-up in 2012, 58 patients refused to continue to participate in the cohort, what could be explained by the lack of information of the 2011 initial group of patients about the annual follow-ups to come. Globally however, when considering patients both recruited in 2011 and in 2012, this amounted to only a tenth of the total baseline sample size. In addition, the comparison of characteristics of participants to the baseline and of participants to the 2013 follow-up did not highlight major differences.

CoDiab-VD will allow drawing a broad picture of patients with diabetes and their care over time. It will also contribute to the evaluation of the PcD and to support decisions about which targeted actions to implement.

## Additional file

Additional file 1:
**Detailed description of the patients’ questionnaire.** A comprehensive description of the patients’ questionnaire regarding the different variables and validated instruments included, as well as their response options or scoring. (DOCX 40 kb)

## Abbreviations

ADDQoL: Audit of Diabetes-Dependent Quality of Life
AUDIT-C: Alcohol use disorders and identification test-consumption
AVD: Association Vaudoise du Diabète
CCM: Chronic Care Model
CDM: Chronic disease management
CHF: Swiss franc
CKD: Chronic kidney disease
CoDiab-VD: Cohort of patients with diabetes in the canton of Vaud
HbA1C: Glycated haemoglobin
HRQoL: Health-related quality of life
MCS: Mental component score
OAD: Oral anti-diabetic drug
PACIC: Patient Assessment of Chronic Illness Care
PcD: Programme cantonal Diabète
PCS: Physical component score
SF-12: 12-Item Short-Form Health Survey
