# Supplementary material for: CoDiab-VD: protocol of a prospective population-based cohort study on diabetes care in Switzerland
Source: BMC Health Serv Res. 2015 Aug 14;15:329. doi: 10.1186/s12913-015-0991-0 (PMC4536695; doi:10.1186/s12913-015-0991-0)
Supplement: Additional file 1: — Detailed description of the patients’ questionnaire. A comprehensive description of the patients’ questionnaire regarding the different variables and validated instruments included, as well as their response options or scoring. (DOCX 40 kb) [file 12913_2015_991_MOESM1_ESM.docx]

**Additional file 1**

**Detailed description of the patients’ questionnaire**

| **Section** | **Themes** | **Instruments, details** | **Response option** |
| --- | --- | --- | --- |
| Diabetes | Characteristics of the disease | Type of diabetes | type 1, type 2, other, do not know |
|  |  | Disease duration | 1-5 years, 6-10 years, 11-15 years, 16-20 years, >20 years |
|  |  | Treatment (drugs) | oral anti-diabetic medications (OAD), insulin, OAD and insulin, none, do not know |
|  | Diabetes-related complications | List of complications | ischemic heart diseases, stroke, retinopathy, chronic kidney disease (CKD) without dialysis, CKD with dialysis or kidney transplant, neuropathies, foot ulcer, lower limb amputation, severe hypo- or hyperglycemia |
|  | HbA1C | Last HbA1C value (among HbA1C-aware patients) |  |
| Diabetes management | Medication adherence | Morisky Medication Adherence Questionnaire [1] | 4 questions and one global score (0=high adherence, 1-2= medium adherence, 3-4=low adherence) |
|  | Receipt of recommended processes-of-care | 1) Past 12 months: |  |
|  |  | HbA1C check (among HbA1C-aware patients) | 1x/year, ≥2x/year, none, do not know |
|  |  | Blood pressure measurement | 1x/year, 2-3x/year, ≥4x/year, none, do not know |
|  |  | Weight measurement | yes, no, do not know |
|  |  | Lipid profile | yes, no, do not know |
|  |  | Diabetic foot examination | yes, no, do not know |
|  |  | Urine test for microalbuminuria | yes, no, do not know |
|  |  | Eye examination by ophtalmologist | <1 year ago, 1-2 years ago, >2 years ago, never, do not know |
|  |  | Influenza vaccination | yes, no, do not know |
|  |  | 2) Anytime: |  |
|  |  | Physical activity recommendations | yes, no, do not know |
|  |  | Diet recommendations | yes, no, do not know |
| Health-related quality of life (HRQoL) and Quality-of-life measures (QoL) | Generic HRQoL | SF-12 [2], physical and mental summary scores (PCS, MCS) | 12 questions and two summary scores (range 0=worst score to 100=best score; scores constructed to have a mean of 50 and a standard deviation of 10 in the general US population) |
|  | Diabetes-specific QoL | ADDQoL [3] | 19 questions and one global score (range -9=maximum negative impact of diabetes to +3=maximum positive impact of diabetes) |
| Patient assessment of chronic care | Congruency of care with the Chronic Care Model (CCM) | PACIC [4, 5] | 26 questions and one global score based on the 20 first questions (range of individual questions and global score: 1=lowest score to 5=highest score) |
| Health services utilisation | Ambulatory care visits | Utilisation during past 12 months: primary care physicians (general internal medicine, family medicine or general practice), diabetologists, nurse specialists, dieticians, podiatrists | 1x, 2-3x, ≥4x, no |
|  | Emergency visits | Utilisation during past 12 months | 1x, 2-3x, ≥4x, never |
|  | Hospitalisation | Utilisation during past 12 months | 1x, >1x, never |
|  | Home care services, domestic home support | Received help during past 12 months | yes regularly, yes temporarily, no, do not know |
|  | Foregoing care because of costs | Foregoing care during past 12 months | yes, no, do not know |
|  |  | If care forgone, please specify (list) | primary care physician consultation, diabetologist consultation, podiatrist consultation, consultation to other healthcare professionals (dieticians, nurse specialists,…), intervention/treatment requiring hospitalisation, medication, dental care, home care, equipment and specialized material |
| Health status and health habits | Anthropometric values | Weight | in kilogram (kg) |
|  |  | Height | in centimeter (cm) |
|  | Smoking | Smoking status | current; former; non smoker |
|  |  | Duration of smoking | years |
|  |  | Smoking products | cigarettes, pipe, cigars, cigarillos, cannabis |
|  |  | Average number of cigarettes smoked per day | 1-9, 10-19, 20-39, >40 |
|  |  | Medical advice on smoking cessation | yes, no, do not know |
|  | Alcohol consumption | AUDIT-C questionnaire [6] | 3 questions and one global score (at risk drinking score ≥3 for women and ≥4 for men) |
|  | Physical activity levels | Questions from the Swiss Health Survey [7] | 4 questions and one global score allowing to classify patients as inactive, partially active, irregularly active, regularly active, trained |
|  | Depression screening | Two validated questions for the screening of depression [8] | patients are considered to be screen-postive for depression if at least one of the two questions are answered positively |
|  | Comorbidities | List of chronic diseases | heart disease (heart failure, valve disease, heart muscle disease), chronic lung disease (asthma, chronic bronchitis, emphysema), osteoporosis, osteoarthritis or arthritis, cancer or malignancy or lymphoma (with the exception of skin cancer), gastric or duodenal ulcer, depression, Parkinson disease, hypertension, hyperlipidemia, other chronic condition |
| Self-management activities and support | Home glucose self-monitoring |  | yes, no |
|  | HbA1C knowledge |  | yes, no, do not know |
|  | Participation in diabetes education classes |  | <1 year ago, 1-2 years ago, >2 years ago; never; do not know |
|  | Membership in the local diabetes association |  | yes, no, do not know |
|  | “Diabetes Passport”* | Knowledge of the “Diabetes Passport” | yes, no, do not know |
|  |  | Use of the “Diabetes Passport” | yes always, yes often, yes seldom, no never |
|  | “Self-efficacy” | Level of easiness/difficulty to manage diabetes generally, and regarding physical activity, diet, and medication | 4 questions, one for each domain (very easy, easy, neither easy nor difficult, difficult, very difficult) |
|  | Information about diabetes | Level of information about diabetes | very well informed, well informed, neither good nor badly informed, badly informed, very badly informed |
|  |  | Source of information | medical doctor; other healthcare professionals – nurse, dieticians, pharmacist…; media – internet, television, paper, radio…; social network; patients’ association or health network; other; none |
|  | Support and satisfaction from healthcare team and social network | Support | one question for the healthcare team and one for the social network (always, often, sometimes, rarely, never) |
|  |  | Satisfaction | one question for the healthcare team and one for the social network (excellent, very good, good, average, poor) |
|  | Diabetes care satisfaction and recommandation of their care to other people | Overall care satisfaction | excellent, very good, good, average, poor |
|  |  | Care recommandation to others | yes definitely, yes probably, no |
| Programme cantonal Diabète† | Activites/projects of PcD | Knowledge | yes, no |
|  |  | Participation | yes, no |
| Socio-demographics | Characteristics of the participants | Age | years (continuous) |
|  |  | Gender | female, male |
|  | Socio-economic status | Marital status | single, married or living with a partner, separated or divorced, widowed |
|  |  | Family size | live alone, live with x persons |
|  |  | Household income in Swiss franc (CHF)/month | ≤3499, 3500-5499, 5500-9499, ≥9500, do not know (i.e quartiles of household income as given by the cantonal office of statistics, for the latest year available) |
|  |  | Education | primary - compulsory school or less, secondary - vocational training or high school, tertiary - university or technical college |
|  |  | Employment | employed full time, employed part time, unemployed or disabled, not in labour force |
|  |  | Health insurance status | ordinary health insurance, specific type of health insurance schemes limiting access to specialist physicians, other, do not know |
|  |  | Supplementary health insurance for hospitalisation | yes, no, no but wanted to (insurance refusal, over expensive premium(s), significant access to care restriction), do not know |
|  |  | Receipt of health insurance subsidies | yes, no, do not know |
|  |  | Place of residence | urban, semi-urban, rural |
|  |  | Nationality | Swiss, European, Extra-European |
| HbA1C = Glycated haemoglobin, HRQoL = Health-related Quality of Life, QoL = Quality of Life, SF-12 = Short Form-12 Health Survey, PCS = Physical Component Score, MCS = Mental Component Score, ADDQoL = Audit of Diabetes-Dependent Quality of Life 19, CCM = Chronic Care Model, PACIC = Patient Assessment of Chronic Illness Care, AUDIT-C = Alcohol Use Disorders and Identification Test-Consumption, PcD = Programme cantonal Diabète  * A small booklet with data, information and reminders.  † Since the 2012 recruitment. | | | |

1. Morisky DE, Green LW, Levine DM: **Concurrent and predictive validity of a self-reported measure of medication adherence**. *Medical care* 1986, **24**(1):67-74.

2. Ware J, Jr., Kosinski M, Keller SD: **A 12-Item Short-Form Health Survey: construction of scales and preliminary tests of reliability and validity**. *Medical care* 1996, **34**(3):220-233.

3. Bradley C, Todd C, Gorton T, Symonds E, Martin A, Plowright R: **The development of an individualized questionnaire measure of perceived impact of diabetes on quality of life: the ADDQoL**. *Quality of life research : an international journal of quality of life aspects of treatment, care and rehabilitation* 1999, **8**(1-2):79-91.

4. Glasgow RE, Whitesides H, Nelson CC, King DK: **Use of the Patient Assessment of Chronic Illness Care (PACIC) with diabetic patients: relationship to patient characteristics, receipt of care, and self-management**. *Diabetes care* 2005, **28**(11):2655-2661.

5. Iglesias K, Burnand B, Peytremann-Bridevaux I: **PACIC Instrument: disentangling dimensions using published validation models**. *International journal for quality in health care : journal of the International Society for Quality in Health Care / ISQua* 2014, **26**(3):250-260.

6. Bush K, Kivlahan DR, McDonell MB, Fihn SD, Bradley KA: **The AUDIT alcohol consumption questions (AUDIT-C): an effective brief screening test for problem drinking. Ambulatory Care Quality Improvement Project (ACQUIP). Alcohol Use Disorders Identification Test**. *Archives of internal medicine* 1998, **158**(16):1789-1795.

7. **Swiss Health Survey** [<http://www.bfs.admin.ch/bfs/portal/fr/index/infothek/erhebungen__quellen/blank/blank/ess/04.html>]

8. Whooley MA, Avins AL, Miranda J, Browner WS: **Case-finding instruments for depression. Two questions are as good as many**. *Journal of general internal medicine* 1997, **12**(7):439-445.
